# Supplementary material for: A ROCK Inhibitor Promotes Graft Survival during Transplantation of iPS-Cell-Derived Retinal Cells
Source: Int J Mol Sci. 2021 Mar 22;22(6):3237. doi: 10.3390/ijms22063237 (PMC8004718; doi:10.3390/ijms22063237)
Supplement: Supplementary file 1 [file ijms-22-03237-s001.pdf]

Supplementary Materials

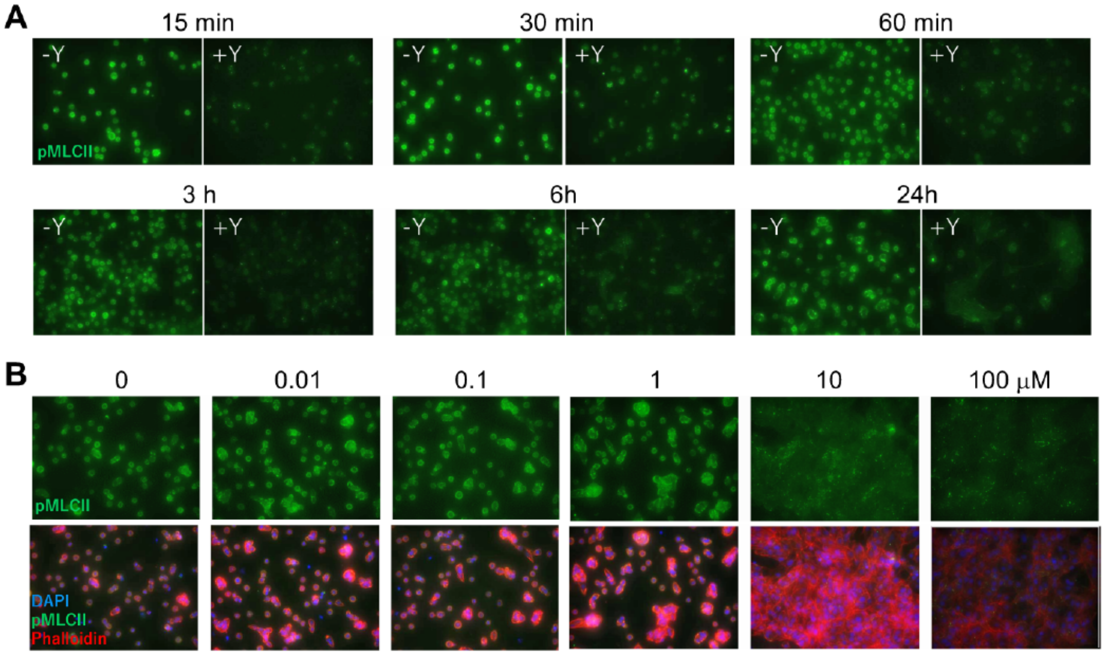

Figure S1. Detection of phosphorylated myosin light chain 2 (pMLCII) in Y-27632-treated iPS-RPE cells. (**A**) IHC for pMLCII (green) in human iPSC-RPE cells without or with 10 μM Y-27632 in timecourse analysis (15 min, 30 min, 60 min, 3 h, 6 h, and 24 h). (**B**) IHC for pMLCII (green) and phalloidin (red)in human iPS-RPE cells with 0, 0.1, 1, 10, and 100 μM Y-27632 in dosage analysis.

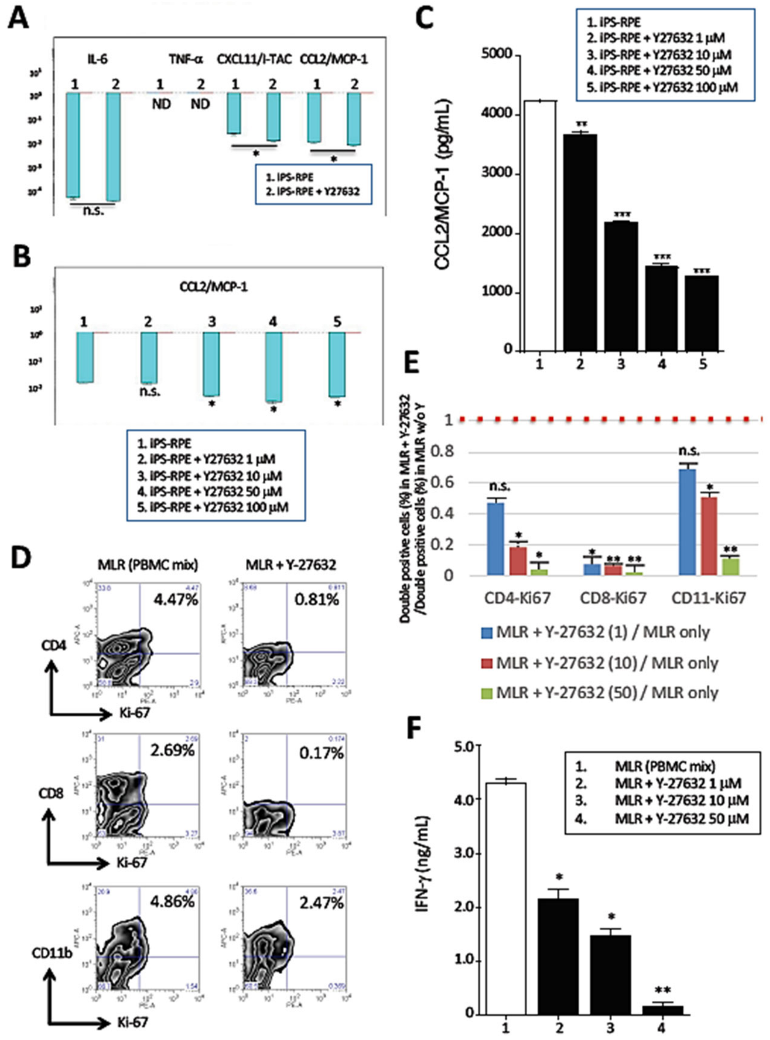

Figure S2. Suppression of production of inflammatory cytokines/chemokines in Y-27632-.treated iPS-RPE cells and capacity of Y-27632 to suppress the activation of lymphocytes *in vitro*. We examined *IL-6, TNF*-a, *CXCL11/I-TAC*, and *CCL2/MCP-1* in Y-27632-treated iPS-RPE cells by qRT-PCR and ELISA. (**A**) In qRT-PCR, Y-27632-treated iPS-RPE cells expressed less mRNA for *IL-6, CXCL11/I-TAC,* and *CCL2/MCP-1*, although the RPE cells did not express mRNA for *TNF*-a. **P* < 0.05 as compared with two group. ND, not detected. n.s. – not significant. (**B**) In a dose-dependent assay with Y-27632 (1, 10, 50, and 100 µM), Y-27632-treated iPS-RPE cells, especially 10, 50, and 100 µM, produced less *CCL2/MCP-1* mRNA compared with non-treated RPE cells. **P* < 0.05 as compared with control culture (non-treated cells). n.s. – not significant. (**C**) We obtained similar results with ELISA for CCL2/MCP-1: Y-27632-treated iPS-RPE cells poorly produced CCL2/MCP-1 protein. ***P* < 0.005, *** *P* < 0.0005 as compared to the control (non-treated RPE cells, open bar). (**D-F**) Capacity of Y-27632 to suppress activation of lymphocytes *in vitro.* We also examined whether Y-27632 directly suppressed the activation of lymphocytes. (**D**) Y-27632 directly suppressed cell proliferation of immune cells: CD4^{+}/Ki-67^{+} (proliferative helper T cells), CD8^{+}/Ki-67^{+} (proliferative cytotoxic T cells), and CD11b^{+}/Ki-67^{+} (proliferative monocytes) when mixed lymphocyte reactions (MLR) were performed by FACS analysis. (**E**) In a dose-dependent assay with Y-27632 (1, 10, 50 µM), Y-27632 greatly suppressed cell proliferation of these lymphocytes. **P* < 0.05, ** *P* < 0.005 as compared with control culture (non-treated MLR). n.s. – not significant. (**F**) Y-27632 significantly suppressed the production of IFNg inflammatory cytokines in Y-27632 and PBMC cocultures.

*$P < 0.05$, ** $P < 0.005$ as compared to the control (PBMC mix without Y-27632, open bar).

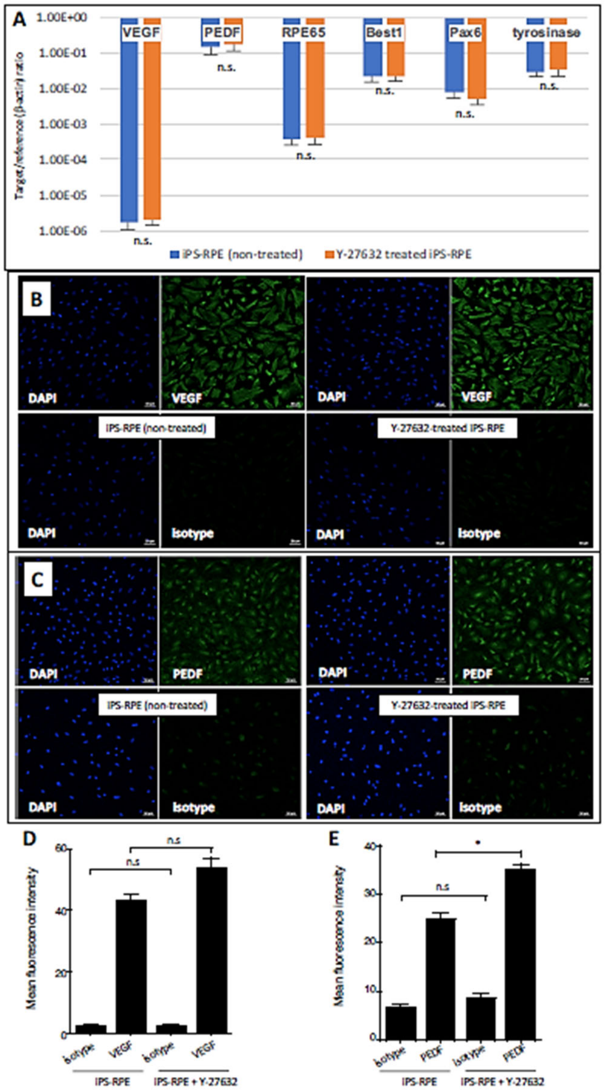

Figure S3. Toxicity of iPS-RPE cells by Y-27632 treatment. We examined the expression of RPE-specific markers such as VEGF-A, PEDF, RPE65, bestropin-1, Pax6, and tyrosinase in Y-27632-treated iPS-RPE cells. (**A**) In qRT-PCR, there were no statistical differences between Y-27632-treated and non-treated RPE cells. (**B**) In IHC, Y-27632-treated iPS-RPE cells and non-treated cells clearly expressed VEGF on their surfaces. Isotype = RPE staining with rabbit IgG. Scale bar in IHC = 50 μm. (**C**) Results for PEDF IHC in Y-27632-treated iPS-RPE cells. Isotype = RPE staining with rabbit IgG. Scale bar in IHC = 50 μm. (**D**) There were no significant differences between Y-27632-treated and non-treated RPE cells in the expression of VEGF. (**E**) However, there were significant differences between Y-27632-treated and non-treated cells in the expression of PEDF, indicating that Y-27632-treated iPS-RPE cells greatly produced PEDF growth factor, which is a RPE specific marker, when compared with non-treated cells. Thus, RPE cells exposed to the ROCK inhibitor experienced no harm or toxicity. * $P < 0.05$ between two groups. n.s., not significant.

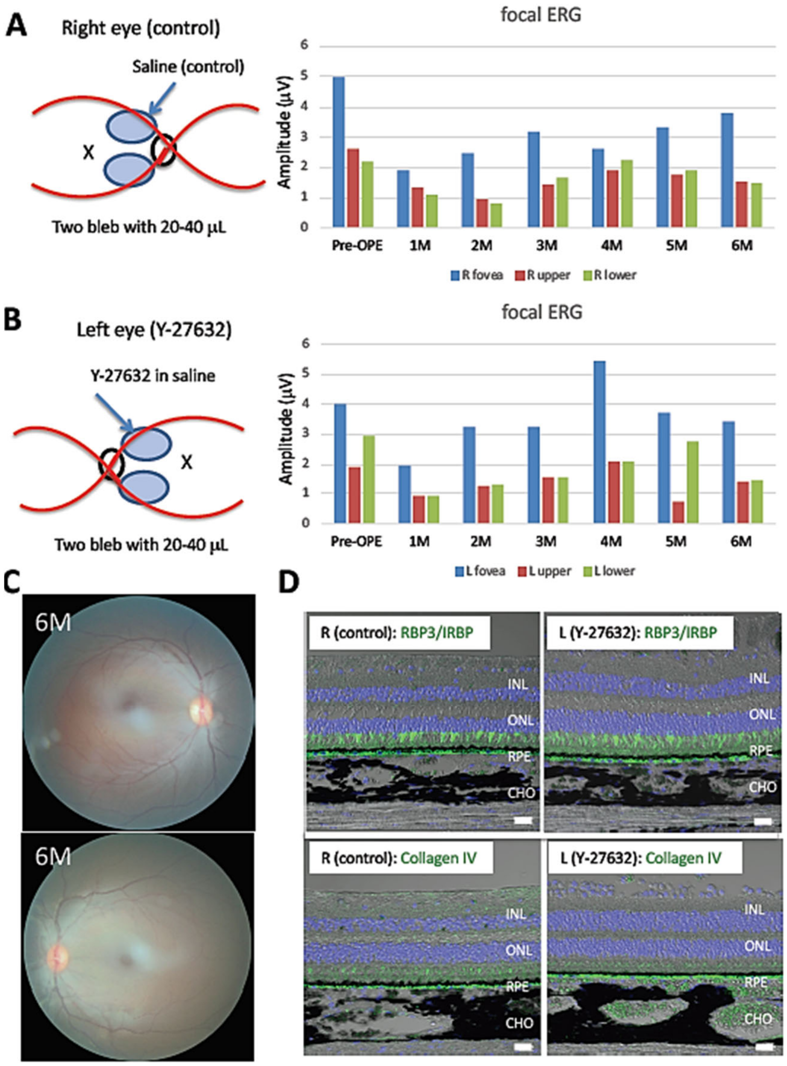

Figure S4. In vivo retinal toxicity in a non-human primate by Y-27632 treatment. We administrated saline only as a control into the right eye (**A**) and Y-27632 in saline into the left eye (**B**) (20-40 μL, respectively) in a normal adult cynomolgus monkey. We then measured focal ERG in the retina of Y-27632 in the left eye and control right eye. Compared with pre-operative data (Pre-OPE), ERG was temporary decreased in both retinas at 1 month after administration, and then the data recovered to baseline at 2-3 months. There were no significant differences between them after a six-month evaluation, indicating that Y-27632 administration in the eye did not change retinal sensitivity. (**C**) Similarly, there were no differences between Y-27632-injected and control retinas in color fundus; 6M, six months. (**D**) In IHC, neural retina & RPE clearly expressed RBP3/IRBP (a marker for neural retina and RPE cells), and the RPE cell layer clearly expressed collagen IV (a marker for basement membrane). These results indicated that there was no retina damage or toxicity after Y-27632 administration in vivo. Scale bar in IHC = 20 μm.

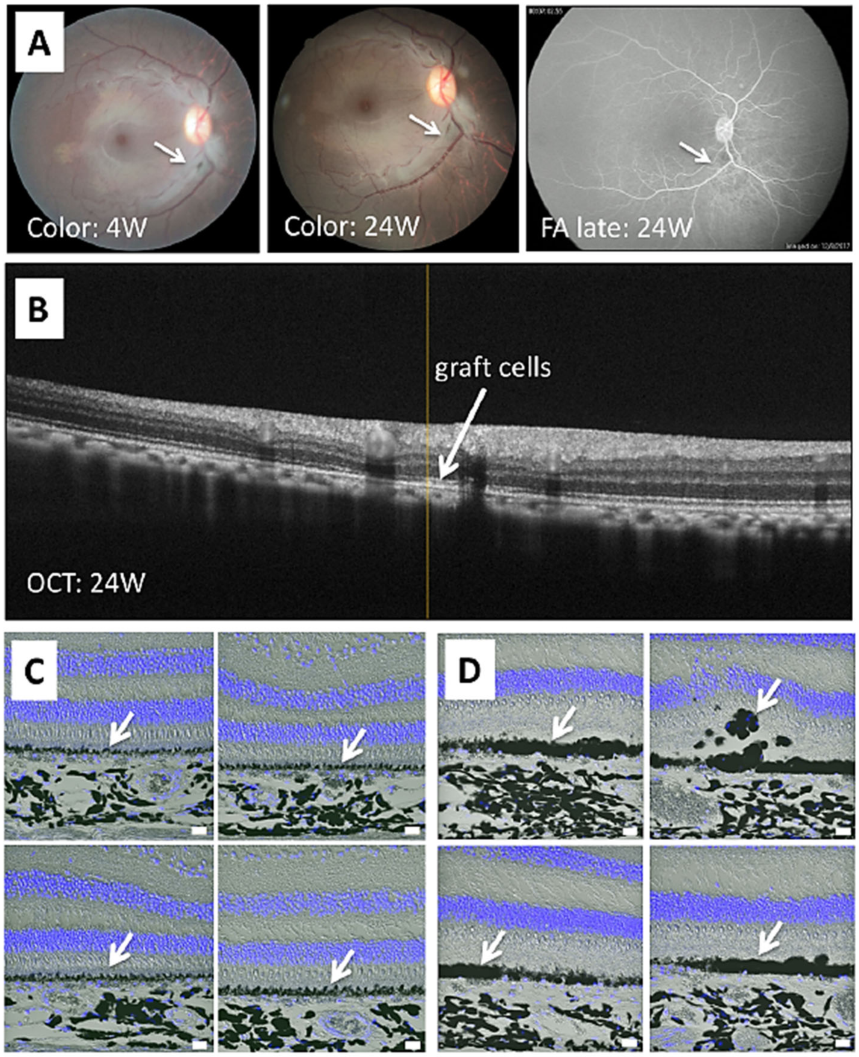

Figure S5. Transplantation of allogeneic iPS-RPE cells in an *in vivo* animal model (without Y-27632, control eye). Without Y-27632, we transplanted allogeneic iPS-RPE cells (46a line) by the same method. (**A**) In color fundus, we found pigmented graft cells, but they were not expanded (clump-like) in the retina of the monkey right eye at 4 and 24 weeks, and there were no abnormal signs such as rejection at 24W FA (right panel). (**B**) We found graft RPE cells without any abnormal signs in the subretinal space at 24W in OCT evaluation. Compared with retinal sections in the right eye without Y-27632 (**C**), the *in vivo* expanded graft RPE cells were seen throughout the retinal sections from the left eye (**D**). Arrows show transplanted graft iPS-RPE cells. Scale bar in IHC = 20 µm.
